# Description and outcome of a cohort of 8 patients with WHIM syndrome from the French Severe Chronic Neutropenia Registry

**DOI:** 10.1186/1750-1172-7-71

**Published:** 2012-09-25

**Authors:** Sarah Beaussant Cohen, Odile Fenneteau, Emmanuel Plouvier, Pierre-Simon Rohrlich, Gerard Daltroff, Isabelle Plantier, Alain Dupuy, Delphine Kerob, Blandine Beaupain, Pierre Bordigoni, Fanny Fouyssac, Anne-Lise Delezoide, Gilles Devouassoux, Jean François Nicolas, Philippe Bensaid, Yves Bertrand, Karl Balabanian, Christine Bellanne Chantelot, Françoise Bachelerie, Jean Donadieu

**Affiliations:** 1 AP-HP, Registre français des neutropénies chroniques sévères, Centre de référence des déficits Immunitaires Héréditaires, Service d’Hémato-oncologie Pédiatrique Hôpital Trousseau, 26 avenue du Dr Netter, 75012, Paris, France; 2 Service d’Onco-Hématologie Pédiatrique, Centre Hospitalo-Universitaire de Besançon, Paris, France; 3 AP-HP, Hôpital Robert Debré Laboratoire d’Hématologie, 75019, Paris, France; 4 Service de Pédiatrie, Centre Hospitalier Belfort-Montbéliard, Paris, France; 5 Service d’hématologie, Centre Hospitalier de Roubaix, Paris, France; 6 Service de Dermatologie, CHU Rennes-Pontchaillou, 35033, Rennes, France; 7 AP-HP Service de Dermatologie Hôpital Saint Louis, 75010, Paris, France; 8 Service de Médecine Infantile II, Hopitaux de Brabois, Vandoeuvre les Nancy Cedex, Paris, France; 9 AP-HP, Service de Biologie du Développement, Hôpital Robert Debré, 75019, Paris, France; 10 Service de Pneumologie, Centre Hospitalier Lyon-Sud, Paris, France; 11 Service d’immunologie clinique et allergologie, Centre Hospitalier Lyon-Sud, Paris, France; 12 Service d’Hématologie Pédiatrique, Hôpital Robert Debré, 75019, Paris, France; 13 Institut d’Oncologie et Hématologie Pédiatrique, Centre Hospitalo-Universitaire Lyon, Paris, France; 14 Inserm UMR_S996, Univ. Paris-Sud, Laboratory of Excellence in Research on Medication and Innovative Therapeutics (LERMIT), 32 rue des Carnets, 92140, Clamart, France; 15 15 AP-HP, Hôpital Pitié-Salpêtrière, Département de Génétique, Univ. Pierre et Marie Curie, 75013, Paris, France

**Keywords:** WHIM, CXCR4, Registry, Neutropenia, Myelokathexis, Infections

## Abstract

**Background:**

WHIM syndrome (WS), a rare congenital neutropenia due to mutations of the CXCR4 chemokine receptor, is associated with Human Papillomavirus (HPV)-induced Warts, Hypogammaglobulinemia, bacterial Infections and Myelokathexis. The long term follow up of eight patients highlights the clinical heterogeneity of this disease as well as the main therapeutic approaches and remaining challenges in the light of the recent development of new CXCR4 inhibitors.

**Objective:**

This study aims to describe the natural history of WS based on a French cohort of 8 patients.

**Methods:**

We have reviewed the clinical, biological and immunological features of patients with WS enrolled into the French Severe Chronic Neutropenia Registry.

**Results:**

We identified four pedigrees with WS comprised of eight patients and one foetus. Estimated incidence for WS was of 0.23 per million births. Median age at the last visit was 29 years. Three pedigrees encompassing seven patients and the fetus displayed autosomal dominant heterozygous mutations of the *CXCR4* gene, while one patient presented a wild-type *CXCR4* gene. Two subjects exhibited congenital conotruncal heart malformations. In addition to neutropenia and myelokathexis, all patients presented deep monocytopenia and lymphopenia. Seven patients presented repeated bacterial Ears Nose Throat as well as severe bacterial infections that were curable with antibiotics. Four patients with late onset prophylaxis developed chronic obstructive pulmonary disease (COPD). Two patients reported atypical mycobacteria infections which in one case may have been responsible for one patient’s death due to liver failure at the age of 40.6 years. HPV-related disease manifested in five subjects and progressed as invasive vulvar carcinoma with a fatal course in one patient at the age of 39.5 years. In addition, two patients developed T cell lymphoma skin cancer and basal cell carcinoma at the age of 38 and 65 years.

**Conclusions:**

Continuous prophylactic anti-infective measures, when started in early childhood, seem to effectively prevent further bacterial infections and the consequent development of COPD. Long-term follow up is needed to evaluate the effect of early anti-HPV targeted prophylaxis on the development of skin and genital warts.

## Background

In 1964, Zuelzer reported an exceptional congenital neutropenia associated with hyperplasia of mature neutrophils in the bone marrow (BM) – *myelokathexis* (retention of white blood cells in the BM) [1]. Its acronym (WHIM) derived from the manifestations of Human Papillomavirus (HPV)-induced Warts, Hypogammaglobulinemia, and bacterial Infections together with Myelokathexis [2]. A marked lymphopenia, which affects both T- and B-lymphocytes and NK cells, completes the picture. The clinical onset and complications in WHIM syndrome (WS) are more variable than originally suspected with the notable exceptions of neutropenia and lymphopenia, which are always observed in patients suffering from this disorder [3]. WS is also genetically heterogenous. Most patients present heterozygous autosomal dominant mutations of the gene encoding for CXCR4, the receptor of the CXCL12 chemokine (or Stromal cell Derived Factor-1) [4], which notably regulates hematopoiesis and peripheral trafficking of neutrophil and lymphocyte subsets. CXCR4 engagement by CXCL12 induces typical activation of Gαi protein-dependent pathways. All *CXCR4* mutations described so far result in partial truncations of the receptor’s carboxyl terminal tail (C-tail), with the exception of the recently described missense non truncating E343K mutation [5], and impair the desensitization process which precludes further G-protein activation thus leading to enhanced and prolonged responsiveness of CXCR4 mutants to CXCL12 (*i.e.* gain of function) [6]. Leukocytes from the minority of patients who carry a wild-type (WT) *CXCR4* gene presented a similar pattern of aberrant CXCL12/CXCR4 responses [4,7]– [9] consistent with a role for these dysfunctions in the WS hematological defects [10]. In support of this assumption, a new knock-in mouse strain that harbors a WS-associated heterozygous mutation of the *CXCR4* gene exhibits striking parallels to the major immunological features of WS (*i.e.* panleukopenia) and is considered as a valuable model of the human syndrome [11].

An exhaustive literature review since the first description in 1964 identified 52 cases originating from the United States, Japan or Europe (Additional file 1) [1,2,4,5,9,12]– [43]. Recurrent infections may be quite severe, but other presentations are more indolent while the white blood cell count (WBC) appears to be affected in a large range, from mild lympho-neutropenia to near panleukopenia. Therefore, the therapeutic management of these patients is diverse. Some patients have no prophylactic therapy, while others may receive prophylactic antibiotics or antiviral therapies such as Immunoglobulins (Ig), Granulocyte macrophage colony-stimulating factor (GMCSF), Granulocyte colony-stimulating factor (GCSF) and eventually undergo hematopoietic stem cell transplantation [30]. Recently, plerixafor (or AMD3100), a small synthetic antagonist of CXCR4 approved for BM hematopoietic progenitor cells transplantation [44], has been tested in WS patients and found to promote the mobilization of neutrophils and lymphocytes to the peripheral blood [31,45]. These studies provide the first pharmacological evidence of the causal role of the gain of CXCR4 function in WS-associated panleukopenia and explain the rationale for the use of CXCL12/CXCR4 antagonists in the management of WS. Both the extreme rarity of WS and its clinical heterogeneity prompted us to analyze all cases identified in France through the national Severe Chronic Neutropenia Registry. We collected the patients’ exhaustive clinical, hematological and biological features, as well as their family histories, and performed the genetic and functional analyses of the CXCR4 receptor.

## Patients and methods

### Organization of the French registry and data monitoring

All patients included in this study were registered in the French Severe Chronic Neutropenia Registry. The registry was created in 1993; since then, enrollment has been prospective. We included all types of congenital neutropenia [46]. The registry received national certification in the year 2008 by the French health authorities. Thirty-five French pediatric hematology-oncology clinical units participate in this registry. Data monitoring was based on the review of medical records collected by a clinical research associate who visited each center yearly, and multiple sources ascertained the completeness of each case. The patient or his legal guardians provided written informed consent before being included in the registry. Several reports of the registry are available elsewhere [47-49].

We used the common definition of WS. Briefly, WS was diagnosed in patients with both neutropenia (defined by at least one absolute neutrophil count (ANC) below 1.5 G/L) and myelokathexis on the BM smear after central review. In addition to this phenotype, we considered *CXCR4* mutations as diagnostic criteria. When available, we screened patients for biological CXCR4 dysfunctions. All these criteria were found in one patient (UPN 5592) who presented no *CXCR4* mutation and whose cytological findings have been previously reported [50]. The patient UPN 5446 has been partially reported elsewhere [51] as well as the functional studies from three individuals of the cohort (UPN 5592, UPN 5446 and UPN 5231) [7,8].

### Phenotyping

We only analyzed routinely recorded parameters such as auxologic status and major medical events requiring medical management. Ig levels were analyzed according to age and expressed as standard deviations (SD) [52]. We obtained systematic recording of infectious episodes from the patients’ written medical history. We defined severe infections as those that would be life threatening without appropriate antibiotic or antifungal therapy and that entail medical supervision or hospitalization. These events were exhaustively registered in the medical records. Minor infections were those for which patients did not seek medical surveillance such as stomatological or ear, nose and throat (ENT) infections. These events were often omitted in the medical records. Warts were considered as a WS manifestation when their number was equal or superior to ten or when they presented as genital *condylomata acuminita*. We performed HPV genotyping for one case of skin warts and one case of ano-genital warts following a Quantitative PCR on frozen biopsies with HPV-specific primers.

### CXCR4 *gene testing*

The patients or their parents gave their written informed consent for genetic and functional testing. Genomic DNA was extracted from blood with standard procedures. The coding sequence and exon-intron boundaries of the *CXCR4* gene (encompassing two exons) were amplified using the primers and PCR conditions described elsewhere. PCR products were sequenced in both directions with the ABI PRISM Big Dye Terminator v1.1 Ready Reaction Cycle Sequencing kit (Applied Biosystems) on an ABI PRISM 3100 Genetic Analyzer (Applied Biosystems). Sequences were analyzed with the Seqscape software v2.2 (Applied Biosystems). We numbered mutations as recommended by the Human Genome Variation Society (http://www.hgvs.org/), using the reference sequence NM_003467.2.

### Functional evaluation of the CXCL12/CXCR4 axis

Studies aimed at investigating the functioning of CXCR4 and other chemokine receptors (CCR5, CCR7, CXCR7) were performed as previously described [7,8].

### Statistical analysis

Stata software version 10 was used for all statistical analyses. Lower and upper interquartile, and median values depict the distribution of quantitative variables. Considering that we ascertained the completion of the enrolment only from 1990 to 2006, we only took into consideration the years 1990–2006 to calculate the epidemiological parameters. Our research extracted the number of births per year in France (metropolitan areas excluding Reunion Island, French Polynesia or French Antilles) from the records of the Institut National de la Statistique et des Etudes Economiques (http://www.insee.fr). Incidence at birth was supposed to satisfy a Poisson distribution. For survival analysis the endpoints were death, and the first episode of cancer. The period taken into account was the time interval from birth to the first date when the event was observed or to the last examination when no event occurred. The Kaplan-Meier method was used to estimate survival rates. The cut-off date was February 1st, 2012. Data from the foetus was not included in the statistical analysis for survival.

### Literature review

In order to identify all publications related to WS, we first screened PubMed, with the key words: myelokathexis and WHIM. We then checked the bibliography of each article in order to identify additional references and to avoid duplicates.

## Results

### Demographic data

We analyzed data from eight subjects (including 6 males) presenting WS and one 32 weeks gestational age medically terminated foetus, originating from four pedigrees (Table 1). The family trees of the four pedigrees are shown in the Additional file 2. The median age at the last follow up was 29 years [p25 8 years – p75 40 years]. Definitive WS diagnosis, as defined by the detection of a *CXCR4* mutation or of the characteristic CXCR4 dysfunctions in leukocytes, was established at the median age of 20 years (SD 25.9) (min 1 year, max 75.6 years), but for the three cases born in the last ten years this diagnosis was made before the age of 2 years, facilitated by an informative family history in two cases and by more readily available *CXCR4* sequencing. WS-linked symptoms first occurred at the median age of 1.3 years (SD 2.32) [min birth- max 5.1 years]. The initial clinical manifestations of WS were bacterial infections for five patients, skin warts for one patient, and isolated neutropenia for two patients. Incidence at birth was 0.23 per million births and the 95% confidence interval limits were 0.0019 – 0.29 per million births.

**Table 1:** Demographic, clinical and genetic data of the patients

**UPN**	**First manifestation Age & type**	**Gender / Age at last visit / Vital status (Life/Death)**	**Warts yes/no Age at first warts comments**	**Genital Warts (HPV vaccine +/−**)	**Infection site: germ (Age)**	**Cancer or malformation**	**CXCR4 mutations DNA / protein**
5546	5.1 years Profuse warts	F/ 32 years / L	Yes 5.1 years surgery at 10 years	No (−)	Urinary tract infection : *E. coli* (23 years)	No	c.969_970insG G323fs343X
5449	6 months Adenitis	M/ 7 years / L	Yes 5 years	No (−)	Adenophlegmon: methicilline-sensitive *S. aureus* (6 months)	No	
					Paroditis: non identified germ (7 years)		
Foetus	NR	F/ Medical termination of pregnancy at 32 gestational weeks/D	NR	NR	NR	Double aortic arch	
5780	1.6 years Salmonellosis	M/ 6.7 years / L	No	No (+)	Digestive infection: *Salmonella typhimurium* (12 months)	No	c.1000C>T R334X
					Osteoarthritis: *S. aureus* (15 months)		
5592	3 months Mastoiditis	M / 24 years / L	Yes 2 years Pharmacoresistance (imiquinod) and reappearance after surgery	No (−)	Repeated pneumonia (since age 7 years) chronic bronchiectasis and lobectomy Repeated otitis media leading to cholesteatoma	No	Wild type
					Stomatitis *HSV1* (12 years)		
					Sinusitis ,Mastoiditis : *Aspergillus glocus* (16 years)		
					Axillary abcess *P. mirabilis* (20 years)		
					Sepsis *H. influenzae* , *S. pneumoniae*		
7012	5-10 years ENT	M/ 75.6 years / L	No	No (−)	ENT (childhood)	Basal cell carcinoma (72 years)	c.1013C>G S338X
					Severe pneumonia (10 years) - chronic bronchiectasis		
					Angiocholitis (age NR)		
5231	At birth Neutropenia	F / 39.5 years / D	Yes Childhood	Genital warts since puberty HPV 6 (−)	Pneumonia (*S. aureus S. pneumoniae, H. influenza, P. aeruginosa),* since childhood leading to chronic bronchiectasis.	HPV related verrucosis metastatic carcinoma of the vulva	
					Chronic sinusitis		
					Genital *HSV2* (23 years)		
					Angiocholitis (27 years)		
					Chronic skin granuloma (nontypable *mycobacterium)* (32 years)		
					Sepsis *P. aeruginusa, Cytomegalovirus (40 years)*		
					Sputum *Candida albicans , P aeruginosa, E. Coli (40 years)*		
					Intestinal tract *C. jejuni, S. aureus , C. albicans* (40 years)		
5446	5-10 years ENT	M /40.6 years / D	Yes Childhood HPV 2, 5,23	Yes (−)	Stomatitis HSV 1 (24 years)	EBV negative cutaneous T-cell lymphoma (37 years)	
					Repeated pneumonia, since infancy complicated by lung abcesses and chronic bronchiectasis : S. *aureus, H. influenzae* , *C. albicans, M. morganii* (33 years), S. *pneumoniae* (34 years), *B. catarrhalis* (35 years), *Proteus mirabilis* (36 years)		
					Purulent pericarditis *Pneumococci* (35 years)		
					Skin lesions *Molluscum contagiosum* and Onycomycosis (NR germ)		
					Atypical *Mycobacteria* hepatitis (40.6 years)		
5447	2 months Neutropenia	M / 8.6 years / L	No	No (+)	No severe infection	Tetralogy of Fallot	

ATB: Antibiotic prophylaxis; CXCR4: Chemokine (C-X-C-motif) receptor type 4; D: Death; DNA: deoxyribonucleic acid; EBV: Epstein Barr virus; ENT: Ears nose throat infection; HPV: Human Papillomavirus; Ig: Immunoglobulin prophylaxis; L: Life, NR: Non reported; PLTs: Platelets; UPN : Unique patient number; + Present; - Absent.

### Bacterial, fungal and mycobacterial infections

Five patients had a history of repeated ENT infections in childhood, whereas two patients (UPN 55476 and 5780) reported less than one ENT episode per year, and another patient treated with prophylactic Ig since the age of 6 months (UPN 5447) reported no ENT episodes. Severe bacterial infections manifested in seven cases as mastoiditis, osteoarthritis, nephritis, angiocholitis, bacterial adenotidis and armpit, submandibulary or pulmonary abscesses. The first episode of severe bacterial infection was recorded at the median age of 10.05 years [min 1.24 years – max 33.7 years]. However, the frequency of these severe bacterial infections was variable; three patients experienced less than one episode every five years. Nevertheless, four patients without a history of overt pulmonary infections developed COPD and bronchiectasis. These were recorded at the age of 8 years for one patient and after the age of 30 years for three other patients. These long-term complications were associated with late anti-bacterial prophylaxis (including Ig and antibiotic rotations). Conversely, the only patient so far free from any infection at the age of 8.6 years was diagnosed with WS at the age of 6 weeks during a pre-operative screening, and has since been maintained on continuous Ig prophylaxis (UPN 5447).

Identified pathogens were *Staphylococcus aureus* (three cases), *Escherichia coli*, *Streptococcus pneumoniae* and *Haemophilus influenzae* (two cases each), *Salmonella typhimurium, Aspergillus glocus, Pseudomonas aeruginosa, Morganella morganii, Moraxella catarrhalis, Proteus mirabilis,* and *Candida albicans (*one case each). Atypical *Mycobacterium* was observed in two patients. In one case, mycobacteria infection was limited to the skin and although the organism was not identified, the referent physician based the diagnosis on the presence of an epithelioid granuloma on a skin biopsy and on the sensibility to rifampicine, isoniazide and pyrazinamid. In the second case, the laboratory identified *Mycobacterium gordonae* in the patient’s sputum. Meanwhile, the patient developed severe liver failure responsible of death. Although the liver biopsy did not show any granuloma, displaying only a non-specific inflammation, we hypothesize that the mycobacteria infection likely contributed to this liver failure.

### HPV-induced warts

Phenotypically, five patients showed varying degrees of HPV-induced lesions. Skin warts generally first appeared on hands or feet, but could also affect the face. They developed for three patients at the ages of 5.1, 5 and 2 years, the date of onset remaining unknown for the other two patients. The three patients exempt from warts were aged 75.6, 8.6 and 6.7 years. There was no association between the age of the skin warts onset and the severity of the wart proliferation. Extensive pharmaco-resistant cutaneous verrucosis that reappeared after surgical ablation severely impaired the quality of life for one patient (UPN 5592).

Besides warts, one male and one female patient reported anogenital *condylomata acuminata* (vulva, vagina, cervix or rectum mucosa). Vulvar and perianal lesions were first recorded at the age of 18 years for the female patient (UPN 5231). In addition, she suffered from recurrent sporadic genital infections by herpes viruses since the age of 23 years, despite oral acyclovir prophylaxis, and aggressive treatment with intravenous valaciclovir and foscarnet. Later, she exhibited intractable multifocal dysplastic HPV-induced lesions despite several years of repeated cervical conizations and extensive surgery (*i.e*., vulvectomy, pelvectomy, colostomy, hysterectomy) and ultimately developed invasive cancer with an early fatal course at the age of 39.5 years. The male patient (UPN 5446), whose numerous skin warts showed HPV serotypes 2, 5 and 23, also presented severe *condylomata acuminata* of the ano-rectal area by the age of 31 years that were surgically removed. We performed HPV serotyping on the anogenital lesions of the female patient (UPN 5231) for low risk types 6, 11, 44, 53 and 54 and high risk types 45, 31, 18 and 16 and 33, and only HPV type 6 was detected. This finding was unexpected considering the lesions’ progression to carcinoma. To try to prevent the development of these mucosal HPV lesions, two of the youngest patients (UPN 5780 and 5447) were vaccinated at 3 and 5 years of age with quadrivalent HPV vaccine (designed against HPV serotypes 6, 11, 16, and 18). Of note, neither patient has presented warts until now.

### Hematological and Immunological parameters

**Figure 1:**
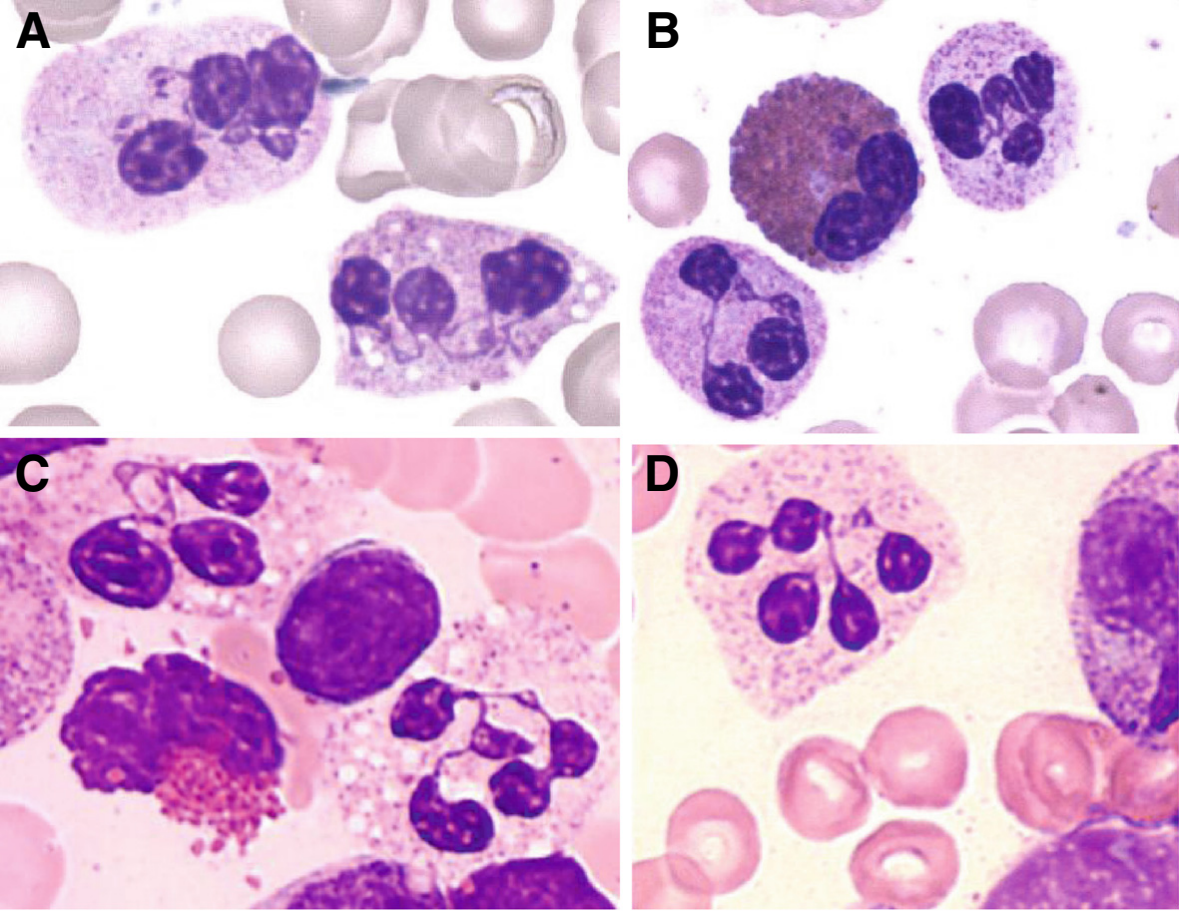
**Bone marrow smears from four patients with myelokathexis, revealing over 50% of neutrophils with abnormal nuclei encompassing 3 to 5 lobes connected by long thin chromatin filaments, and less than 10% vacuolated mature neutrophils visible on images A and C.** (May-Grunwald-Giemesa stain; original magnification x 1000). **A**, patient UPN 5780; **B**, patient UPN 5446; **C**, patient UPN 5546; **D**, patient UPN 5449.

At the time of WS diagnosis, median WBC was 1.050 G/L (p25: 0.7 - p75: 3.7 G/L), ANC was 0.23 G/L (p25: 0.2 - p75: 0.4 G/L), absolute lymphocyte count (ALC) was 0.63 G/L (p25: 0.42 - p75: 0.7 G/L), and absolute monocyte count (AMC) was 0.09 (p25: 0.083 - p75: 0.24 G/L). The median hemoglobin (Hb) level was 11.5 g/dL (p25: 10.2 g/dL - p75 12 g/dL) and platelet count was 201 G/L (p25 172 - p75 223 G/L). Transient anemia (< 7 g/dL) was observed in two patients, and transient mild thrombocytopenia (between 50 and 120 G/L) was observed in three patients, without obvious etiology in both cases. During routine follow-up, a median of 16 baseline complete blood cell count (CBC) values per patient was available. For all cases, WBC and ANC fluctuated with time, without any detectable regular variation or significant change observed over time, with the exception of the transiently normalized blood count values recorded during infectious episodes. The Additional file 3 illustrates the dynamic variation of the WBC of four patients at the occasion of sepsis or of GCSF therapy. Taking into account all the available CBC recorded during the routine follow up of the patients (Table 2), with a median number of 17 per patient, the median WBC was 1.1 G/L (p25: 0.85 - p75: 1.7 G/L), the ANC was 0.19 G/L (p25: 0.15 - p75: 0.37 G/L), the ALC was 0.63 G/L (p25: 0.0.42 - p75: 0.7 G/L), and the AMC was 0.1 (p25: 0.09 - p75: 0.13 G/L). The median Hb level was 11 g/dL (p25: 10.6 g/dL - p75 12.1 g/dL) and the median platelet count was 212 G/L (p25 181 - p75 336 G/L). Seven BM smears were available for central review, all showing rich BM: the granulocytic lineage represented a total of 36% of BM cells, with 18.5% mature granulocytes, of which 2.75% were monocytes and 23% were lymphocytes. Examination confirmed myelokathexis in all cases (Figure 1). Four patients were treated with 5 μg/kg/day GCSF resulting in a significant increase in the peripheral ANC from a median of 0.340 × 10^9^/L before treatment, to a maximum median of 1.499 × 10^9^/L under GCSF. AMC and ALC were not significantly modified (0.11 versus 0.10 G/L and 0.57 versus 0.35 × 10^9^/L, respectively before and under GCSF treatment). The total duration of the GCSF treatment was extremely short in one patient (13 days). The three patients who received long term GCSF therapy, interrupted treatment in the absence of significant efficiency to prevent recurrent infections. Indeed, for patient UPN 5446, despite three years of apparent normalization of the ANC consecutive to daily Lenograstim subcutaneous injections, the number of infectious pulmonary episodes was not diminished. The registry recorded similar findings for patients UPN 5231 and 5592.

**Table 2:** Hematological, immunological features and summary of medical management

**UPN**	**ANC**	**ALC**	**AMC**	**PLTs**	**Ig G**	**Ig A**	**Ig M**	**CD3**	**CD4**	**CD8**	**CD19**	**GCSF**
	**×10**^**9**^**/L**	**×10**^**9**^**/L**	**×10**^**9**^**/L**	**×10**^**9**^**/L**	**g/L**	**g/L**	**g/L**	**×10**^**9**^**/L**	**×10**^**9**^**/L**	**×10**^**9**^**/L**	**×10**^**9**^**/L**	**ATB**
												**Ig**
												**Other**
5546	0.16	0.38	0.08	223				0.33	0.2	0.1		No GCSF
												No ATB
												No Ig
5449	0.16	2.08	0.16	452	11.6 (6 mo)	0.12	0.199	1.33	0.89	0.35	0.16	No GCSF
												No ATB
												No Ig
Foetus	NR	NR	NR	NR	NR	NR	NR	NR	NR	NR	NR	NR
5780	0.16	1.27	0.1	367	2.83*	0	0.53	1	0.77	0.13	0.07	GCSF short course / efficient
												No ATB
												Early Ig+
5592	1.32	0.3	.021	60	-	0.8	0	0.1	0.02	0.02	0.09	GCSF since age 5 years, on demand since age 8 years / repeated infections despite GCSF :
												ATB+
												Ig +
												Warts treated by surgery and imiquinod with no efficiency
7012	0.22	0.58	0.09	202	9.69	1.89	<0.25	0.67	0.30	0.42	0.01	No GCSF
												No ATB
												No Ig
5231	0.4	0.17	0.07	212	4.2	0.5	0.4	0.3	0.04	0.04	0	GCSF since age 28 years /repeated infections despite GCSF
												No ATB but Aciclovir prophylaxis
												Ig +
5446	0.34	0.42	0.12	193	7.2	0	0	0.33	0.15	0.19	0.04	GCSF since age 34 years / no effect on bronchitis Withdrawn
												ATB+
												Ig +
5447	0.14	1.2	0.1	306	1.8**	1.0	0	0.6	0.54	0.04	0	No GCSF
												No ATB
												Ig +

ALC: Absolute lymphocyte count; AMC: Absolute Monocyte count; ANC: Absolute neutrophil count; GCSF: Granulocyte colony stimulating factor; Ig: Immunoglobulin prophylaxis; NR: Non reported; PLTs: Platelets; UPN : Unique patient number; + Present; - Absent **Antibody against vaccine: normal titers.

The total number of lymphocytes was extremely low among patients with median CD3+ T cells determined at 0.465 × 10^9^/L (SD 0.41) (min 0.1, max 1.33), median CD4+ T cells at 0.25 × 10^9^/L (SD 0.33) (min 0.02, max 0.89), median CD8+ T cells at 0.11 × 10^9^/L (SD 0.14) (min 0.02, max 0.42), and median CD19+ B cells at 0.02 × 10^9^/L (SD 0.05) (min 0, max 0.16). Despite the low total lymphocyte count*,* the proportion of lymphocyte subsets was apparently preserved (Table 2).

We evaluated vaccine antigen responses in two patients. Patient UPN 5780 showed neither anti-tetanus nor anti-*H*. *influenzae* b antibodies (Abs) despite vaccination 6 months prior, whereas the patient UPN 7012, who had been vaccinated more than ten years ago, presented low anti-tetanus Abs, normal anti-pneumococcal Abs, but no anti-poliovirus Abs. Ig levels were evaluated in seven patients. IgG levels were mildly low (between −2 and −3 SD) in two subjects and normal in four subjects. IgA and IgM levels were low in five subjects and normal in two subjects. Five patients received monthly Ig replacement therapy including two children since the diagnosis of WS, and three adults who were also treated with GCSF because of severe recurrent bacterial infections. Prophylactic Ig therapy seemed efficient in preventing bacterial infections, particularly in the younger patients of the cohort, who did not present any further severe bacterial infection since the introduction of Ig prophylaxis. Interestingly, three patients who received neither Ig nor GCSF were free of any bacterial infection.

### Genetics

We screened *CXCR4* mutations for all the patients including the foetus: seven patients among three families presented mutations of the *CXCR4* gene and one patient had a WT *CXCR4* gene (Table 1). We found a new mutation 969_970insG in two related patients, a C_1013_→G substitution in three patients from one pedigree, and a C_1000_→T substitution in one patient (respectively, in the CXCR4 proteins G323fsX343, S338X and R334X). These nonsense or frameshift mutations led to C-tail truncations of the CXCR4 protein.

### Long term outcome and associated pathology

Patient UPN 5446 developed cutaneous T-cell lymphoma of the right ankle at the age of 37 years uncorrelated with an Epstein Barr virus (EBV) infection. His father, UPN 7012, presented basal cell carcinoma at the age of 72 years. Two premature deaths were reported in two siblings respectively at 39.5 and 40.6 years. The causes of death were metastatic vulvar carcinoma and possible *Mycobacteria*-related liver failure. Severe cardiac conotruncal malformations were present in two subjects from unrelated pedigrees with different *CXCR4* mutations (Table 1). The first case was diagnosed with Tetralogy of Fallot (TOF) at birth and was successfully operated at the age of two months. The second was diagnosed *in utero* with a double aortic arch that led to the medical termination of the pregnancy at 32 gestational weeks. Fertility seemed unperturbed in WS patients. One woman gave birth to two children with non-complicated pregnancies, had one miscarriage and one terminated pregnancy. The second woman bore no children. Among affected men, two of the three adult males had children.

## Discussion

In this study we describe the French national cohort of patients with WS, thereby adding 8 patients and 4 pedigrees to the 52 previously reported distinct cases originating from 32 pedigrees (Additional file 1). The genetic studies performed in 27 pedigrees find that 25 pedigrees (including 3 from this report) bear *CXCR4* mutations. R334X, the most frequent mutation, is reported in 15 pedigrees (Table 3). The S338X and S339fs342X mutations are respectively detected in 3 and 2 pedigrees, while the other mutations (E343X, G336X, S341fsX365, the previously unreported G323fs343X mutation herein described and the missense mutation E343K) are reported only once. This survey underlines the extreme rarity of the WS disease, as the birth ratio observed in France can be estimated to 0.23 cases per million births. This incidence rate has been calculated for the period 1990–2006. Nevertheless, we cannot formally exclude the possibility that there may exist some individuals, most likely sporadic cases, with milder clinical courses that do not yet require medical attention and who have therefore not been included in the registry. This low incidence rate, although never calculated worldwide, is in accordance with the scarcity of reported cases as shown in the Additional file 1.

**Table 3:** **Literature summary of *CXCR4 *gene mutations reported in WHIM Syndrome pedigrees – information from 27 informative pedigrees including the two WT *CXCR4***

**Nucleotide change**	**Amino acid change**	**Number of reported pedigrees (including present study)**	**Reference**
c.969_970insG	G323fs343X	1	Present study
c.1000C>T	R334X	15	[4,43,59,64]– [66] and present study
c.1006G>T	G336X	1	[66]
c.1013C>G	S338X	3	[7,21,62] and present study
c.1016-17delCT	S339fsX342	2	[4,63]
c.1021delT	S341fsX365	1	[41]
c.1027G>T	E343X	1	[4]
c.1027G>A	E343K	1	[5]
Wild Type	No mutation	2	[7,9,32] and present study,

This cohort offers new information about the hematological and infectious profiles of WS. Interestingly, besides the constant neutropenia and lymphopenia, all 8 patients present monocytopenia and half of the patients present profound monocytopenia below 0.1 G/L, contrasting with the monocytosis commonly observed in other congenital neutropenias, such as the elastase neutrophil expressed (ELANE) syndrome. Of particular note, susceptibility to mycobacterial infections may be added to the infection spectrum of WS. Considering the monocytopenia and the infectious profile, composed by pyogenic infections, warts and mycobacteria, WS presents certain similarities with the Mono-MAC syndrome, now identified as the consequence of *GATA2* mutations [53,54]. The major phenotypic difference between the two syndromes is the BM myelokathexis feature the WS.

We previously reported, in leukocytes derived from two patients (UPN 5231 and 5446) from pedigree 4 carrying a mutated CXCR4 receptor, that the increased Gαi protein-dependent signaling (*e.g.* CXCL12-induced chemotaxis) was associated with the inability of CXCR4 to be uncoupled from G proteins (*i.e.* desensitized) and internalized in response to CXCL12 [7], which is in agreement with other studies (reviewed in [55,56]). Functional studies could not be assessed in two patients (UPN 5546 and 5449) from pedigree 1 who did not give their consent. In this genetic form of WS, impaired CXCR4 desensitization and internalization resulted from distal truncations of the receptor’s C-tail thereby removing potential phosphorylation sites involved in the attenuation process. Interestingly, we also described a similar pattern of CXCR4 dysfunctions in leukocytes from one subject carrying a WT *CXCR4* open reading frame and myelokathexis (UPN 5592) with a full WS phenotype (pedigree 3). This suggests that altered CXCL12/CXCR4-mediated signaling is a common biological trait of WS caused by different genetic mutations, and that for the patients with a WT *CXCR4*, the genetic cause(s) may involve an effector protein of the CXCL12/CXCR4 axis [7]. Later, fibroblasts and EBV-immortalized B cells derived from this patient were found to display dysfunctions of G protein coupled receptor kinase 3 (GRK3) pointing to the key role of this kinase in the regulation of the CXCR4 receptor attenuation [8]. However, the genetic anomalie(s) causing the GRK3 dysregulation, and the subsequent impairment in CXCR4 inactivation, remain unknown.

Defining WS with certitude is a difficult exercise in the absence of identified *CXCR4* mutations since none of the terms of the WS acronym (*W*arts *H*ypogammaglobulinemia *I*nfections and *M*yelokathexis) have full sensibility or specificity. Warts concern 58% of the cases in literature and in agreement with this frequency, five out of eight cases in our cohort. Hypogammaglobulinemia is usually very mild with reported levels between −1 and −2 SD and rarely below. Accordingly, in this cohort, patients presented variable IgG levels with no cases exhibiting frank hypogammaglobulinemia below −3 SD. Although infection susceptibility is on the contrary almost always present, severe infections are rare. Finally, myelokathexis is not pathognomomic of WS, as it is observed in other situations such as in neutropenia linked to the glucose 6 phosphatase, catalytic subunit 3 (*G6PC3)*[57], *CXCR2* loss-of-function mutations [58] or in gastric cancer [59]. In addition, for three patients from this study, BM smears were initially described as showing only an absence of myeloid blockage, while we identified the typical multi-lobular nuclei of myelokathexis only at the time of the central review. Moreover there is one described WS case with documented *CXCR4* mutation but no myelokathexis (P6 [41]). On the contrary, the constant panleukopenia and monocytopenia [3,56,60] are not included in the acronym of the WS. This panleukopenia is also reported in the literature. Indeed, taking into account all original publications displayed in the Additional file 1, the median lowest WBC is 1.65 (mean 1.96; p25: 1 - p75: 2.60 G/L), the median lowest ANC is 0.33 (mean of 0.46; p25: 0.13 - p75: 0.75), with constant lymphopenia and deep monocytopenia (< 0.2 G/l). According to these observations we now suggest WILM (*W*arts, *I*nfections, *L*eukopenia, and *Myelokathexis*) as a new acronym of the syndrome.

To make the definitive diagnosis of WS in the absence of any *CXCR4* mutation, we propose that the association of these ‘WILM’ features together with the gain of CXCR4 function are needed. Under such conditions, two pedigrees with WT *CXCR4* have been described, one originating from Slovenia [9,15], and one from our survey, for which a *GATA2* mutation was excluded. In addition to these findings, we have studied two patients with *GATA2* mutations who presented WS-like features (*Mycobacterium avium* infection, Warts and Neutropenia) together with an impaired CXCR4 internalization [7,8,61]. This suggests some eventual interplay between CXCR4-dependant signalling and GATA2 that could account for the manifestations of neutropenia and warts of the GATA2 syndrome [62] and beyond, of the more recently described Serine threonine kinase 4 (STK4)-linked syndrome [63].

Of particular note was the apparent paradox between the profoundly altered WBC and the relatively indolent clinical presentations of WS both in our cohort and in the literature. Indeed, ANC, ALC (including both T- and B-cell subpopulations) and AMC are close to those observed in Severe Combined Immunodeficiency Disease patients. However, the spectrum of infections presented by WS patients is rather limited: they only exhibit a higher susceptibility to bacterial infections from encapsulated Gram-positive and -negative bacteria, staphylococcus and mycobacteria. Among the eleven published cases, for which the bacterial pathogens responsible for infection were identified (Additional file 1), *Streptococcus pneumonia* was reported in seven cases, *Haemophilus influenza* in six cases, *Staphylococcus aureus* in three cases, and *Proteus mirabilis*, *Clostridium perfringens* and *Pseudomonas aeruginosa* in one case each. Finally, apart from two reported cases of herpes infections, one case of severe chicken pox [13], one case of rubella and two cases of influenza [5], WS patients mainly present a particular susceptibility to HPV infection that manifests as cutaneous (hand/feet/face) and ano-genital mucosal lesions which abnormally often progress to cancerous lesions. Although we cannot dismiss the hypothesis that the WS immunodeficiency eventually affects anti-HPV immune responses, we rather favor the possibility that the CXCL12/CXCR4 axis represents a host susceptibility factor for HPV-infection and associated carcinogenic evolution [64]. Low risk HPVs that infect the mucosa (such as HPV-6 and −11) do not usually cause cancer in the general population, yet develop as severe dysplasia and carcinoma in WS patients. It must further be pointed out that certain types of infection are not reported in WS such as pulmonar pneumocystosis, zona or severe flu. Thus, the apparent paradox of WS patients is to exhibit a profoundly altered immune function and yet a limited susceptibility to viral and bacterial pathogens, with the notable exception of HPV. This paradox may be explained if we consider that the disease is mainly related to the neutropenia, which, in contrast to other congenital neutropenias, is likely due to an impaired release from the BM that can be transiently overcome during infection, thus resulting in normalization of BM cytology and peripheral neutrophil counts [15,42,43].

**Figure 2:**
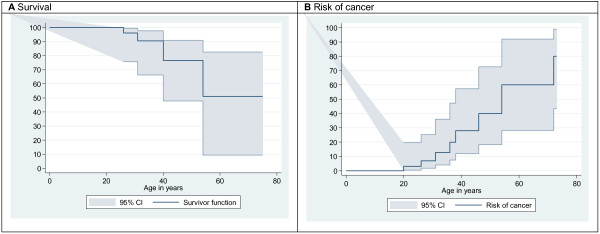
Graphical representation of survival (A) and of the risk of cancer (B) after pooling the 52 published cases of patients with WHIM Syndrome and the 8 cases reported in this survey.

The long-term outcome of WS patients from both our survey and the 52 previously reported patients indicated five deaths (Figure 2A). No death was reported before the age of 20 years with the exception of the medically terminated foetus described in this study. In addition to the two patients herein reported, whose deaths were caused by HPV-induced genital cancer and liver failure, the causes of deaths were lymphoma in two cases (at the ages of 26 [28] and 54 years [41]) and bacterial meningitis in one case (at the age of 31 years [2]). Three other malignancies were reported including one patient who declared a non-lethal B lymphoma at the age of 31 years followed by a maxillary carcinoma [19], and another patient who developed a genital cancer of unspecified etiology at age of 20 years [5]. The overall cancer risk in this population is estimated to be about 30% by the age of 40 years, with an age of onset beyond the third decade (Figure 2B). Genital warts, which are reported for 23% of the literature cases and for 28.6% of cases herein described (2 cases), mark a turning point in the natural course of the disease due to their likelihood to progress to intractable multifocal dysplasia and invasive cancer with an early fatal course despite repeated aggressive surgical removal.

Lastly, the association between WS and TOF has been recently reported [65], which included the patient UPN 5447, reported as P3. This association is strengthened by the herein reported fetus, aborted at 32 gestational weeks due to a double aortic arch, a cardiac abnormality belonging to the same group of conotruncal malformations as TOF.

Even though WS is quite rare, the amount of data collected until now can help to determine the medical management of afflicted patients. The current standard therapeutic approach often associates oral antibiotic prophylaxis, Ig and GCSF. The benefit of Ig prophylaxis is obvious when proposed early in life, as was the case for the two youngest patients of this cohort, but less evident when started later in life, notably after the development of permanent lung damage due to previous infections. The use of GCSF is questionable. Undoubtedly, neutropenia is corrected by GCSF, yet infections do not seem correlated directly to the ANC, and no chronic stomatitis is reported. In addition, patients in our cohort withdrew from GCSF due to the lack of efficiency of the treatment against infections and this pattern was equally frequent in the literature [40]. Long term prophylactic rotational antibiotic therapy is recorded in two patients with COPD (Table 1) in addition to their Ig prophylaxis which seemed to lower the incidence of subsequent pulmonary infection episodes. However, although prophylactic measures can be beneficial on bacterial infections they have no impact on HPV-related disease.

Moreover, for the management of HPV-induced cutaneous and genital warts, standard methods such as cauterization and laser therapy seem relatively inefficient [34]. Aggressive surgical removal, Interferon or Cidofovir administration, as well as topical Imiquimod treatments all proved equally unsatisfying, as lesions tended to reappear. This stresses the need for a better understanding of the physiopathologic mechanisms accounting for the susceptibility to HPV. Prophylactic quadrivalent HPV vaccine administration to a WS patient was recently tested and was associated with protective immunity [25] suggesting that such a vaccine, if its long-term activity can be demonstrated, could be used as early prophylactic treatment for young WS patients and for newly diagnosed WS cases. Hematopoietic stem cell transplantation has been proposed once, but with regards to the natural history of the disease, it seems difficult to propose a pre-emptive program for all patients, as the benefit expected by a transplant is to prevent infections in the third or fourth decade.

We should stress that although the actual management of WS is prophylactic, treatments that specifically target the CXCL12/CXCR4 axis are currently being evaluated. Indeed, two independent phase I clinical trials based on the acute administration of plerixafor have provided the pharmacological evidence of the causal role of CXCR4 dysfunctions in the WS-associated panleukopenia. However, whether such treatment may help to treat or to prevent HPV lesions, EBV lymphoma or *Mycobacteria* infection, remains to be investigated. Moreover, considering that the management of WS will need efficient and safe in the long run chronic treatments aimed at normalizing, but not abolishing the CXCR4 signaling, and because the long-term safety of AMD3100 may be questioned [66,67], alternative new inhibitory compounds need to be characterized. The pre-clinical mouse model of the WS [66,67] will be useful for such analyses.

## Abbreviations

Abs: Antibodies;ALC: Absolute lymphocyte count;AMC: Absolute monocyte count;ANC: Absolute neutrophil count;BM: Bone Marrow;CBC: Complete blood count;COPD: Chronic obstructive pulmonary disease;C-tail: Carboxyl terminal tail;CXCR4: Chemokine (C-X-C motif) receptor type 4;CXCL12: Chemokine (C-X-C motif) ligand 12;ELANE: Elastase neutrophil expressed;EBV: Epstein Barr virus;GCSF: Granulocyte colony-stimulating factor;GMCSF: Granulocyte macrophage colony-stimulating factor;GRK3: G-protein coupled receptor kinase 3;G6PC3: Glucose 6 phosphatase catalytic subunit 3 gene;g.w.: Gestational weeks;Hb: Hemoglobin;HPV: Human Papillomavirus;Ig: Immunoglobulins;PCR: Polymerase chain reaction;SD: Standard deviation;STK4: Serine threonine kinase 4 syndrome;TOF: Tetralogy of Fallot;UPN: Unique patient number;WBC: White blood cell count;WS: WHIM (Warts Hypogammaglobulinemia Infections Myelokathexis) syndrome

## Competing interests

The authors declare no competing interests.

## Authors’ contributions

OF carried out the bone marrow central review, CBC performed the molecular genetic studies, KB and FB were responsible for the CXCR4 functional studies and analyses. EP, PSR, GD, IP, AD, DK, PB, FF, ALD, GD, JFN, PB, and YB who are referent physicians for the patients, revised the manuscript and contributed to the acquisition and analysis of data with SBC, JD and KB. BB visited each centre yearly to update the register. FB, SBC and JD wrote the manuscript. All authors read and approved the final manuscript.

## Supplementary Material

Additional file 1 **Literature review of 52 published cases of WHIM syndrome.** ** Antibody against vaccine: Normal titers; ALC: Absolute lymphocyte count; AMC: Absolute Monocyte count; ANC: Absolute neutrophil count; ATB: Antibiotic prophylaxis; CXCR4: Chemokine (C-X-C-motif) receptor type 4; EBV: Epstein Barr virus; F: Female, ENT: Ears nose throat infection; GCSF: Granulocyte colony stimulating factor; GMCSF: Granulocyte macrophage colony-stimulating factor; HPV: Human Papillomavirus; Ig: Immunoglobulin prophylaxis; M: Male; NA: Non applicable; NR: Non reported. UPN: Unique patient number; SP: Streptococcus pneumoniae; HI: Haemophilus influenzae; SA: Staphylococcus aureus; PM: Proteus mirabilis; PA: Pseudomonas aeruginosa; NR: Not reported. + Present; - Absent. Click here for file

Additional file 2 Family Trees of the four WHIM syndrome pedigrees described in this study, consistent with autosomal-dominant inheritance and sporadic cases. Click here for file

Additional file 3 Sequential variation of blood counts in four patients illustrating the dynamic of variations of ANC (absolute neutrophil count), ALC (absolute lymphocyte count) and AMC (absolute monocyte count), at the occasion of sepsis and 5μg/kg/day Granulocyte colony-stimulating factor (GCSF) therapy. Click here for file
