# Supplementary material for: Description and outcome of a cohort of 8 patients with WHIM syndrome from the French Severe Chronic Neutropenia Registry
Source: Orphanet J Rare Dis. 2012 Sep 25;7:71. doi: 10.1186/1750-1172-7-71 (PMC3585856; doi:10.1186/1750-1172-7-71)
Supplement: Additional file 1 — Literature review of 52 published cases of WHIM syndrome. ** Antibody against vaccine: Normal titers; ALC: Absolute lymphocyte count; AMC: Absolute Monocyte count; ANC: Absolute neutrophil count; ATB: Antibiotic prophylaxis; CXCR4: Chemokine (C-X-C-motif) receptor type 4; EBV: Epstein Barr virus; F: Female, ENT: Ears nose throat infection; GCSF: Granulocyte colony stimulating factor; GMCSF: Granulocyte macrophage colony-stimulating factor; HPV: Human Papillomavirus; Ig: Immunoglobulin prophylaxis; M: Male; NA: Non applicable; NR: Non reported. UPN: Unique patient number; SP: Streptococcus pneumoniae; HI: Haemophilus influenzae; SA: Staphylococcus aureus; PM: Proteus mirabilis; PA: Pseudomonas aeruginosa; NR: Not reported. + Present; - Absent. [file 1750-1172-7-71-S1.doc]

Additional File 1

Supplementary Table 1a

| Article | Publication year  (several if duplicate)  Country | **Gender** | Age (years) at last update  Death /cause of death | CXCR4 Mutation | Skin Warts  Age onset (years)  Cervical Warts  Chronicity if informed | Myelokathexis | WBC (x10^9^/L) | ANC (x10^9^/L) | ALC (x10^9^/L) | AMC (x10^9^/L) | IgG/A/M (g/L) | Infection Sites | Germs | Cancer or malformation or other | GCSF or GMCSF | ATB | Ig | | Other | |
| --- | --- | --- | --- | --- | --- | --- | --- | --- | --- | --- | --- | --- | --- | --- | --- | --- | --- | --- | --- | --- |
| [1,29] | 1964  USA | F | 10 | NR | No | yes | 28.7 during infection  2.1-2.7 at baseline | 0.12-1.2 at baseline  Intermittent variations | About 1.2 – 1.8 at baseline | 0.04-0.5 | NR | Lungs, bronchiectasis  lymphadenitis | SA | No | No | No | No | | No | |
| [18,33] | 1977  1997  USA (Hawaï) | F | 30 | NR | No | yes | 10 at 10 years old  1.4 at 30 years old | 0.196 | 1.03 | 0.05-0 | At 10 years: IgG 560  IgA 126  IgM 148 | Recurrent pneumonia, ENT | HI  SP | No | No | Yes penicillin | No | | no | |
|  |  | M | 1 |  | No | yes | 2.7-3.2 | 0.2-0.3 | 2.4-2.7 | 0.05 | No | Otitis media, upper respiratory infection | NR | No | no | no | no | | No | |
| [4,9,15,16] | 1981  1992  2000  2003  Slovenia | F | 43 | Wild type | No | yes | NR | 0.39 | NR | NR | NR | Numerous | NR | No | GM-CSF /GCSF | NR | no | | no | |
|  |  | F | 9 |  | No | yes | NR | 0.52 | NR | NR | NR | Acute tonsillitis at age 2 | NR | No | No | No | No | | no | |
| [35] | 1988  Italy | M | 5 | NR | No | yes | 11.2 but rose to 30 with infection | 0.2-1.1 | NR | NR | NR | Lungs, urinary tract infections, otitis, enteritis | NR | Bone ichtyosis vulgaris. Right lung atelectasia | no | no | no | | no | |
| [20,26] | 1989  1992  Germany | F | 22 | NR | Several plantar warts  Recurrent vaginal wall condylomata acuminata | yes | 1.2 | 0.02 | 0.9 | 0.18 | NR | Recurrent cutaneous and upper respiratory infections | NR | No | GM-CSF / GCSF | no | No | | | Interferon alpha 2 condylomata |
| [2,31,32,43] | 1977  1981  19902011  USA | F | 44 | R334X | Skin warts on face, limbs Since age 5  Cervical dysplasia | yes | 1 | 0.27 | 0.65 | 0.07 | IgG 568  IgA 62  IgM 28  ** | Ear, sinus, lungs  bronchiectasis | HI  SA  PM | No | 6 day GM-CSF | no | Yes | | | plerixafor |
|  |  | F | 22 |  | Skin warts on hands, Condylomata acuminatum and dysplasia | yes | 1 | 0.13 | 0.75 | 0.11 | IgG 386  IgA 56  IgM 39 ** | Sinus, ear, lungs | HI  SA | No | 6 day GM-CSF | no | | no |  | |
|  |  | Father M | 31 Died of meningitis and septicemia |  | warts on forearms and hands, | NR | 0.97 | 0.4 | 0.5 | 0.05 | IgG:240-500  IgA:0-60  IgM:0.37 ** | Lungs, meningitis | NR | No | no | no | | no | no | |
| [42] | 1991  USA | F | 11 | NR | No | yes | 2.400 | 0.33 | 1.56 | 0.5 | Normal | Omphalitis, sepsis, bacterial tracheitis, pneumonia, gingivitis, labial cellulitis, periectal abcesses, otitis media, stomatitis. | Clostridium perfringens, PA. | No | G-CSF  5μg/kg 28 days | no | | NR |  | |
| [21] | 1994  South Africa | M | 16 | NR | Skin warts  Age 8 years  Persisted. | yes | 1.9 | 0.9 | 0.456 | NR | At age of 4  IgG 436  IgA 33  IgM 71 | otitits media, cellulites, lungs meningitis. | HI  Severe chicken pox | No | no | no | Yes IM then IV (since age 4) | | no | |
| [27] | 1997  USA | F | 27 | E343X | Skin warts  From age 3 years to mid adolescence. | yes | Initially 0.6 | 0.75 | NR | NR | At age of 15  IgG 670  IgA 0  IgM ** | Lungs : beginning at 3 months bronchiectasis | Not isolated | NR | G-CSF therapy at age 15 for 19 months | yes | | NR |  | |
|  |  | M | 3.25 |  | No | yes | NR | 0.33-0.87 | NR | NR | At age of 3.25  Ig G 497 | Pneumonia  Chronic otitis media | Not isolated. | No | G-CSF 5μ/kg/day | NR | | NR |  | |

Supplementary Table 1b:

| Article | Publication year  (several if duplicate)  Country | **(number if several in the same report) Gender** | Age (years) at last update  Death /cause of death | CXCR4 Mutation | Skin Warts  Cervical Warts  Age onset (years)  Chronicity if informed | Myelokathexis | | WBC (x10^9^/L) | ANC (x10^9^/L) | ALC (x10^9^/L) | AMC (x10^9^/L) | Ig G/A/M (g/L) | Infection Sites | Germs | Cancer or malformation or other | GCSF or GMCSF | | | ATB | Ig | | Other |
| --- | --- | --- | --- | --- | --- | --- | --- | --- | --- | --- | --- | --- | --- | --- | --- | --- | --- | --- | --- | --- | --- | --- |
| [38,39] | 1999  2005  Japan | F | 11 | R334X` | Skin warts on hands and legs  Age 9 | Yes | | 2 | 0.48 | 1.26 | 0.26 | At age of 5  IgG 405  IgA 21  IgM 191 | Recurrent respiratory infections since at age 6 months | HI,  SP | Tetralogy of Fallot | GCSF for 24 h. | | | NR | NR | |  |
|  |  | F | 11 |  | Skin warts on hands and legs  Age 9 | Yes | | 1.4 | 0.06 | 1.22 | 0.06 | At age of 5  IgG 458  IgA 29  IgM 190 | Lungs | HI  SP | Diabetes mellitus at 7 y | GCSF 24h, | | Cotrimoxazole started at 3 years effective | | NR | |  |
| [13] | 2000  USA | F | 40 | NR | No | Yes | | 1 | 0.1-0.3 | NR | NR | At age of 12:  IgG 525 | Pneumonia age 5  Gingivitis, cutaneaous and sinopulmonary infections  Leg ulcer | NR | No | GCSF at 36y (short course) | | | NR | NR | |  |
|  |  | M | 20 |  | No | Yes | | NR | 0.1-0.5 | NR | NR | NR | Otitis media and otitis externa, severe chicken pox, gingivitis, pneumonia, cellulitis | NR | No |  | | | age 4-8  IM IG | NR | |  |
|  |  | F | 20 | NR | Skin warts on hands | Yes | | 0.3-0.7 | < 0.2 | NR | NR | NR | Ear sino pulmunary infections since childhood | NR | No | 3 days GCSF | | | NR | NR | |  |
|  |  | F | 1 |  | NO | yes | | NR | Leuko neutropenia since birth | NR | NR | NR | NR | NR | No | GCSF 3ug/kg/j | | | NR | NR | |  |
| [22]  III3[17,19]  III5 [19] | 2000  2001  2010 | F ( II 2) | 73 | R334X | Skin warts  Age 20  Cervical warts | NR | | 2.3 | 0.59 | NR | NR | NR | Pneumonia  Pansinutis | NR | No | No | | | No | No | |  |
|  |  | M (III 3) | 40 |  | Skin warts Disseminated | NR | | 1.1 | 0.06 | NR | NR | NR | Pneumonia  Pansinutis  Sub cutaneous abcess | NR | At 31 years EBV B cell lymphoma  At 40 years squamous cell carcinoma of the maxillary sinus | Yes before chemotherpary short course | | | No | No | Chemotherapy for B cell lymphoma | |
|  |  | F (III 5) | 46 |  | Skin warts  Age 7  Vulvar warts | NR | | 2.2 | 0.24 | NR | NR | NR | Pneumonia  Pansinutis | HPV 16 | cutaneous basal cell carcinomas, vulvar carcinoma in situ, oral squamous cell carninoma | | No | | NR | NR | |  |
|  |  | M(III 7) | About 30 years |  | Skin warts  Age 5 | NR | | 3.4 | 1.02 | NR | NR | IgG 826  IgA 224  IgM 30 | Cellulitis  Pneumonia  Chronic ear infection  periondotonpathy | NR | NR | NR | | | NR | NR | |  |
|  |  | M (IV 1) | 3 |  | Skin warts  Age of 0.33 | NR | | 1.2 | 0.054 | NR | NR | NR | Impetigo cellulitis | NR | No | No | | | No | No | |  |
|  |  | F (IV 2) | 4 |  | Skin warts lower lip, arms, fingers  Age 4  Genital warts: labia majora, vagina | | NR | 1.3 | 0.18 | NR | NR | NR | Cellulitis | NR | No | No | | | No | No | |  |

Supplementary Table 1c:

| Article | Publication years  (several if duplicate)  Country | **Gender (number if several in the same report)** | Age (years) at last update  Death /cause of death | CXCR4 Mutation | Skin Warts  Cervical Warts  Age onset (years)  Chronicity if informed | Myelokathexis | WBC (x10^9^/L) | ANC (x10^9^/L) | ALC (x10^9^/L) | AMC (x10^9^/L) | Ig G/A/M (g/L) | Infection Sites | Germs | | Cancer or malformation or other | GCSF or GMCSF | ATB | Ig | Other |
| --- | --- | --- | --- | --- | --- | --- | --- | --- | --- | --- | --- | --- | --- | --- | --- | --- | --- | --- | --- |
| [28] | 2002  Japan | F | 26 Death B lymphoma | NR | Skin warts: hands and fingers  Age : childhood.  Generalized skin molluscum contagiosium | yes | NR | NR | NR | NR | At age 6  Ig G 400  Ig A 55  Ig M 82 | Early diagnosis childhood due to frequent infections, pneumonia, axillary adenopathy | Herpes simplex | | EBV+ T / B lymphoma | GCSF since age 13 in case of infections | No | No | Chemotherapy for Lymphoma |
| [40] | 2005  UK | F | 52 | R334X | Skin warts both hands, refractory to treatment,  First seen at age 10 then spontaneous remission.  Vulvar condyloma | yes | NR | 0.1-0.8 | CD3 0.115 | NR | IgG 650  IgA37  IgM 38 | Frequent respiratory sepsis in childhood  Extensive cellulitis of the right leg in childhood  Pneumonia  * | Zoster  HPV  HSV  SP | vulval dysplasia at 33 y /. Evolved in 7 years to vulvar-anal intraepithelial neoplasia | | GCSF 5 ug/kg did not improve clinical condition and had little apparent effect on neutrophil counts. | acyclovir | IVIG very effective |  |
| [12,30] | 2010  2007  Hungary | F | 9  (HSCT at age of 8 y) | p.S338X | No | Yes | From 0.6 to 2.1 G/l | ANC 0.1-0.96G/l in infection free periods | 1.150 with 81% CD3 44% CD4 12CD8 |  | Mild hypogamma  At year of 6  IgG 402  IgA 43  IgM 45 with vaccinal antibodies | Recurrent upper respiratrory tract infections (6 episodes od pneumonia) between age 1 and 6.5 years.  Bronchiectasis, dental periostis | NR | | No | GCSF at 6.5 y |  | IVIG 400mg/kg/month | HSCT  at age 8 |
| [36] | 2006  Sweden | F | 26 | S339fs342X | Skin warts on hands  Appeared at age 7 | yes | NR | NR | Low B and T, low IgG (normal IgM) neutropenia | NR | NR | Repeated urinary tract infections (age 2-7) Recurrent paranasal sinus and lung infections  Recurrent respiratory tract infections | NR | | no | GCSF | no | IVIG therapy high dose age 10 | No |
| [24] | 2007  USA | M | 23 | R334X | Skin warts  Present from age 8 to 10 years and spontaneously resolved until recurrence at age 20 years. | Yes | NR | 0 | NR |  | IgG3 level reduced but IgG, IgA, IgM normal  Vaccinal antibodies normal | Cutaneous abscesses, recurrent otitis media in childhood only | NR | | NO | No | No | No |  |
| [37] | 2008  Poland | F | 27 | R334X | No skin warts Vaginal warts at age 20 | yes | 0.86 | 0.172 | 0.662 |  | At 20 years  IgG 430  IgA37  IgM38 | Recurrent sino-pulmunary infections  HPV | NR | | No | GM and G CSF at age 9 | yes | IVIG |  |
|  |  | M | 0.10 |  | No | Not performed |  | 0.03 | 2.0 |  | At 8 months  IgG 167  IgA0  IgM 17 |  | NR | | Flat angioma | GCSF | yes | Ig IV |  |
|  |  | M | 0.03 |  | No | Not performed |  | 0.031 | 2.852 |  | At 4 months  IgG 250  IgA 7  IgM 41 |  | NR | | No | GCSF | yes | Ig IV |  |

Supplementary Table 1d:

| Article | Publication years  (several if duplicate)  Country | **Gender (number if several in the same report)** | Age (years) at last update  Death /cause of death | CXCR4 Mutation | Skin Warts  Age onset (years)  Cervical Warts  Chronicity if informed | Myelokathexis | WBC (x10^9^/L) | ANC (x10^9^/L) | ALC (x10^9^/L) | AMC (x10^9^/L) | Ig G/A/M (g/L) | Infection Sites | Germs | Cancer or malformation or other | | GCSF or GMCSF | ATB | Ig | other |
| --- | --- | --- | --- | --- | --- | --- | --- | --- | --- | --- | --- | --- | --- | --- | --- | --- | --- | --- | --- |
| [23,41]  P3 already reported [14] | 2004  1984  2009  Italy | (P1) F | 7 | R334X | no | yes | 1.5 | 0.3 | 1.005 | NR | Ig G 415  Ig A12  Ig M33 | Recurrent respiratory tract infections | NR | Tetralogy of Fallot | | 4 (suspended) | yes | − |  |
|  |  | (P2) F | 17 | G336X | yes | yes | 0.4 | 0.32 | 0.312 | NR | Ig G 174  Ig A15  Ig M 125 | Meningitis, recurrent respiratory tract infections | NR | − | | − | Yes | 1 |  |
|  |  | (P3) F | 36 |  | yes | yes | 1.3 | 0.416 | 0.793 | NR | Ig G 438  Ig A 25  Ig M 42 | Recurrent respiratory tract infections | NR | − | | − | No | − |  |
|  |  | *(P4) M* | 14 | R334X | no | yes | 0.72 | 0.079 | 0.482 | NR | Ig G 597  Ig A135  Ig M 60 | Bronchopneumonia | NR | − | | 9.5 | No | 11-12 |  |
|  |  | *(P5) M* | 15 | S341fsX365 | yes | yes | 2.9 | 0.377 | 2.320 | NR | Ig G 375  Ig A42  Ig M 87 | Fever, osteitis | NR | − | | − | No | 3-6 |  |
|  |  | *(P6) F* | 4.5 | R334X | no | No | 1.49 | 0.075 | 1.222 | NR | Ig G 192  Ig A8  Ig M 50 | Enteritis, recurrent respiratory tract infections | NR | Idiopathic mental retardation | | 0.8 | Sporadically | 2 |  |
|  |  | (P7) F | 27 | S338X | Skin warts  Genital condyloma acuminata | yes | 0.8 | 0.208 | 0.401 | NR | Ig G 1137  Ig A243  Ig M 256 | Pneumonia | NR |  | | 27 | yes | − |  |
|  |  | *(P8) F* | 54 Death Lymphoma |  | yes | NA | NA | NA | NA | NR | Ig G 955  Ig A288  Ig M 86 | NA | NR | B cell Lymphoma | | NA | NA | NA |  |
|  |  | *P9* | 25 | S339fsX342 | yes | yes | 0.510 | 0.150 | 0.300 | NR | Ig G NA  Ig A65  Ig M 162 | Protracted enteritis | NR | No | | 25 | sporadically | yes |  |
|  |  | ***(P10) F*** | 9 | R334X | yes | NA | 1.120 | 0.232 | 0.726 | NR | Ig G 790  Ig A130  Ig M 160 | Otitis media, bronchopneumonia, varicella, recurrent respiratory tract infections | NR | − | | − | yes | − |  |
| [34] | 2010  USA | M | 29 | R334X  (Dr Tharp- non published data) | Skin warts hands, Genital warts and perianal warts. | NR | NR | NR | NR | NR | Normal | recurrent pneumonia, leukopenia, and anemia | SP  HPV types 2, 11 (hand) type 6 (genital) | | No | No | No | No | no |
| [25] | 2010  Austria | F | 12 | R334X | No warts | Yes | NR | <1 | NR | NR | NR | Pneumonia, urinary tract infections | NR | No | | No | No | yes | Gardasyl immunization |
| [31] | 2011  USA | F | 30 | R334X | NR | NR | NR | NR | NR | NR | NR | NR | NR | No | | NR | NR | NR | plerixafor |
|  |  | M | 51 | R334X | NR | NR | NR | NR | NR | NR | NR | NR | NR | No | | NR | NR | NR |  |
| [5] | 2012 USA | P1 M | 9 | E343K | Skin warts: multiple clusters on hands near the finger tips and on face; Age 3 | Yes | 2.57 | 1.06 | 1.23 | 0.20 | Ig G 694  Ig A 170  Ig M 70 | respiratory tract, including pharyngitis, sinusitis, pneumonia, and otitis. | Influenza virus | No | | No | NR | NR | NR |
|  |  | P2 F | 5 |  | No warts | Unknown | 3.39 | 1.05 | 1.98 | 0.27 | Ig G 791  Ig A 95  Ig M 72 |  | Influenza virus  SP | No | | yes | yes | NR | NR |
|  |  | P3 F | 34 |  | Skin warts  Genital HPV | Unknown | 3.07 | 1.4 - 1.95 | 0.91 | 0.16 | Ig G 705  Ig A 103  Ig M 113 |  | Rubella virus | No | | No | NR | NR | NR |
|  |  | P4 F | 53 |  | No skin warts  Possible genital warts. | Unknown | 2.93 | 1.45 | 1.19 | 0.24 | Ig G 725  Ig A 103  Ig M 71 | No recurrent infection history | none | Hysterectomy (age 20) unspecified malignancy | | No | NR | NR | NR |

** Antibody against vaccine: Normal titers; ALC: Absolute lymphocyte count; AMC: Absolute Monocyte count; ANC: Absolute neutrophil count; ATB: Antibiotic prophylaxis; CXCR4: Chemokine (C-X-C-motif) receptor type 4; EBV: Epstein Barr virus; F: Female, ENT: Ears nose throat infection; GCSF: Granulocyte colony stimulating factor; GMCSF: Granulocyte macrophage colony stimulating factor; HPV: Human Papillomavirus; Ig: Immunoglobulin prophylaxis; M: Male; NA: Non applicable; NR: Non reported. UPN: Unique patient number; SP: *Streptococcus pneumoniae*; HI: *Haemophilus influenzae*; SA: *Staphylococcus aureus*; PM: *Proteus mirabilis; PA: Pseudomonas aeruginosa;* NR: Not reported. + Present; - Absent.
