# Supplementary figures and images for: Description and outcome of a cohort of 8 patients with WHIM syndrome from the French Severe Chronic Neutropenia Registry
Source: Orphanet J Rare Dis. 2012 Sep 25;7:71. doi: 10.1186/1750-1172-7-71 (PMC3585856; doi:10.1186/1750-1172-7-71)

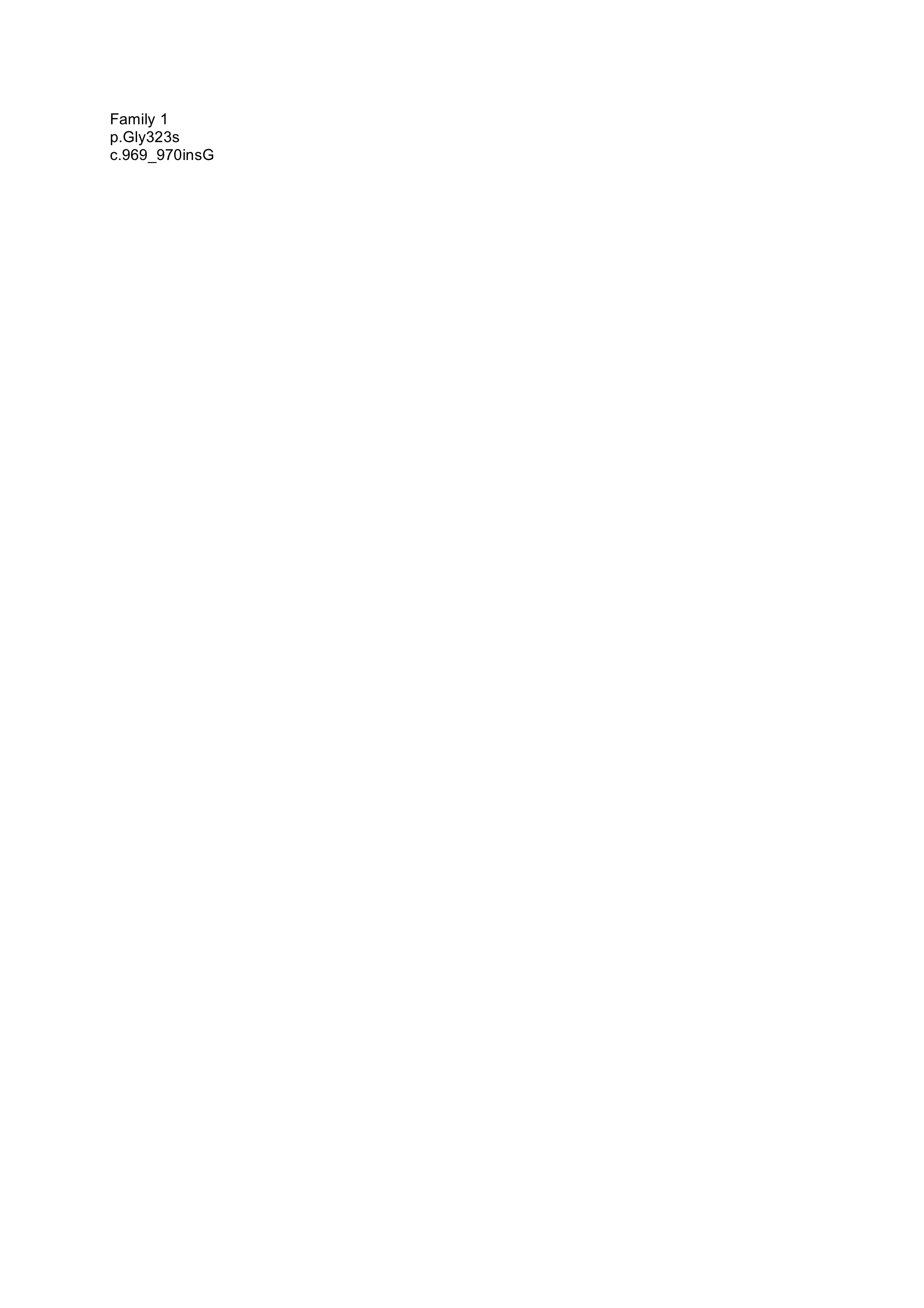


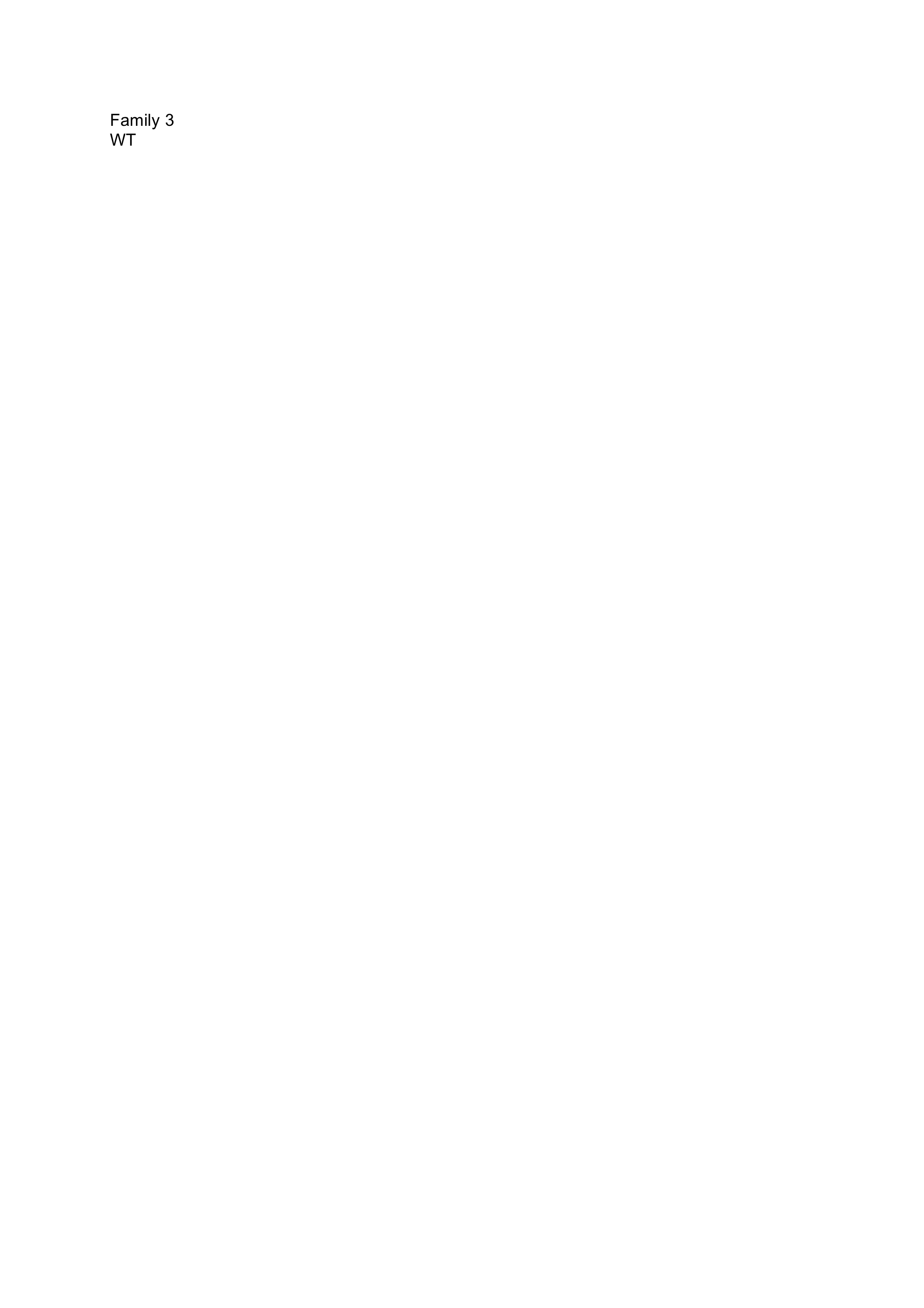


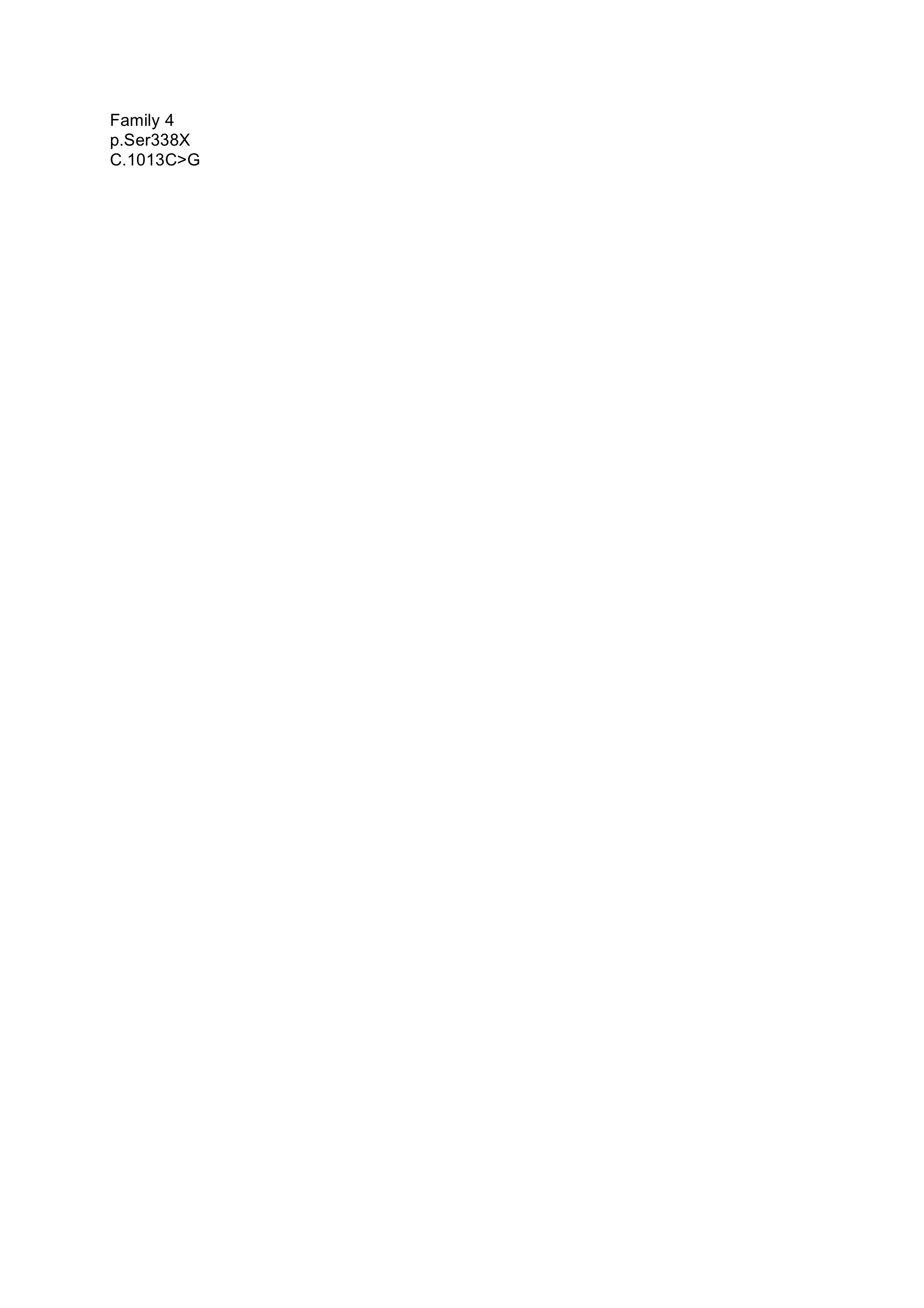

Supplement: Additional file 2 — Family Trees of the four WHIM syndrome pedigrees described in this study, consistent with autosomal-dominant inheritance and sporadic cases. [file 1750-1172-7-71-S2.doc]
