# Supplementary material for: Description and outcome of a cohort of 8 patients with WHIM syndrome from the French Severe Chronic Neutropenia Registry
Source: Orphanet J Rare Dis. 2012 Sep 25;7:71. doi: 10.1186/1750-1172-7-71 (PMC3585856; doi:10.1186/1750-1172-7-71)
Supplement: Additional file 3 — Sequential variation of blood counts in four patients illustrating the dynamic of variations of ANC (absolute neutrophil count), ALC (absolute lymphocyte count) and AMC (absolute monocyte count), at the occasion of sepsis and 5μg/kg/day Granulocyte colony-stimulating factor (GCSF) therapy. [file 1750-1172-7-71-S3.doc]

Additional File 3

|  |  |
| --- | --- |
|  |  |
